# Forecasting influenza-like illness trends in Cameroon using Google Search Data

**DOI:** 10.1038/s41598-021-85987-9

**Published:** 2021-03-24

**Authors:** Elaine O. Nsoesie, Olubusola Oladeji, Aristide S. Abah Abah, Martial L. Ndeffo-Mbah

**Affiliations:** 1 Department of Global Health, Boston University School of Public Health, 801 Massachusetts Ave, Crosstown Center 3rd Floor, Boston, MA 02119 USA; 2 Department of Epidemiological Surveillance, Ministry of Health, Yaoundé, Cameroon; 3 Department of Veterinary Integrative Biosciences, College of Veterinary Medicine and Biomedical Sciences, Texas A & M University, Texas, USA

**Keywords:** Infectious diseases, Computational science

## Abstract

Although acute respiratory infections are a leading cause of mortality in sub-Saharan Africa, surveillance of diseases such as influenza is mostly neglected. Evaluating the usefulness of influenza-like illness (ILI) surveillance systems and developing approaches for forecasting future trends is important for pandemic preparedness. We applied and compared a range of robust statistical and machine learning models including random forest (RF) regression, support vector machines (SVM) regression, multivariable linear regression and ARIMA models to forecast 2012 to 2018 trends of reported ILI cases in Cameroon, using Google searches for influenza symptoms, treatments, natural or traditional remedies as well as, infectious diseases with a high burden (i.e., AIDS, malaria, tuberculosis). The R^2^ and RMSE (Root Mean Squared Error) were statistically similar across most of the methods, however, RF and SVM had the highest average R^2^ (0.78 and 0.88, respectively) for predicting ILI per 100,000 persons at the country level. This study demonstrates the need for developing contextualized approaches when using digital data for disease surveillance and the usefulness of search data for monitoring ILI in sub-Saharan African countries.

## Introduction

Influenza and other respiratory tract infections remain a global public health issue in spite of vaccine availability^1^. Every year, there are about 650,000 deaths globally and three to five million respiratory illnesses due to the influenza virus^2^. In Africa, acute respiratory infections are thought to be a major cause of morbidity and mortality^3,4^, despite considerable under-reporting and sparsity of precise assessments of the total health and economic burden across the continent^3,5^.

The 2009 influenza pandemic reinforced the necessity for developing robust influenza surveillance systems globally^6^. Studies published right after the pandemic noted that many countries in Africa were not equipped with sufficient data on the epidemiology and risk factors of influenza to put in place the necessary approaches for influenza prevention and control^7,8^. This gap in global early-detection and response measures has motivated extensive research towards improving influenza surveillance using both traditional and non-traditional data streams^9–19^, as well as the development of a global standard for influenza surveillance by the World Health Organization (WHO)^20^. Though influenza surveillance data from Africa has improved in recent years, data quality is still lagging behind those implemented in other regions of the world. This lagging is mainly due to limited capacity for laboratory confirmation of influenza diagnoses and timely reporting of virological and epidemiological data. From a global perspective, understanding influenza dynamics in the tropics is important for pandemic planning and response since poor surveillance capabilities can delay the detection of novel viruses^7,21^. Although more than a billion people are living in tropical regions, there are no sufficient data on influenza-related mortality and morbidity^21^.

Digital epidemiology has enabled the use of non-traditional data sources (e.g., social media, Internet search queries) to monitor the spread of influenza-like illness (ILI)^9,10,16,22–25^. However, to our knowledge, no studies have focused on applying digital epidemiology to ILI surveillance in a sub-Saharan African country using data collected by a national surveillance system. Here, we seek to demonstrate that the adoption of digital data for public health surveillance in African countries requires a contextualized approach^26,27^. For example, cultural and societal practices unique to the sub-Saharan African context demands a rethinking of what search terms should be included in models used for surveillance. An individual with influenza-like symptoms might search for terms such as, “cold” or “catarrh”, but might also search for traditional or natural remedies and diseases such as HIV, TB and malaria, which have a high burden across sub-Saharan Africa and are well-known.

In this paper we aim to model and nowcast (hereafter referred to as, forecast) ILI in Cameroon using data from a surveillance system for influenza and ILI implemented by the Ministry of Health (MOH) in Cameroon^28^. Recent studies on influenza and ILI surveillance in Cameroon have focused on viral identification^29^, understanding genetic diversity^30,31^, and seasonality of circulating viruses^32^. Here, we focus on providing a framework for forecasting future trends, which could be invaluable for public health planning and response. Specifically, we considered a range of robust regression modeling approaches that have been previously used for forecasting influenza trends^11,12,33^: random forest regression, support vector regression, multivariable linear regression and ARIMA models. We fitted the models to six years of weekly reported ILI cases and google search data and used one year of data, not used for model fitting, for model cross-validation and comparison. The modeling approaches with the best fit were then used in one-week ahead forecasting.

## Results

**Figure 1:**
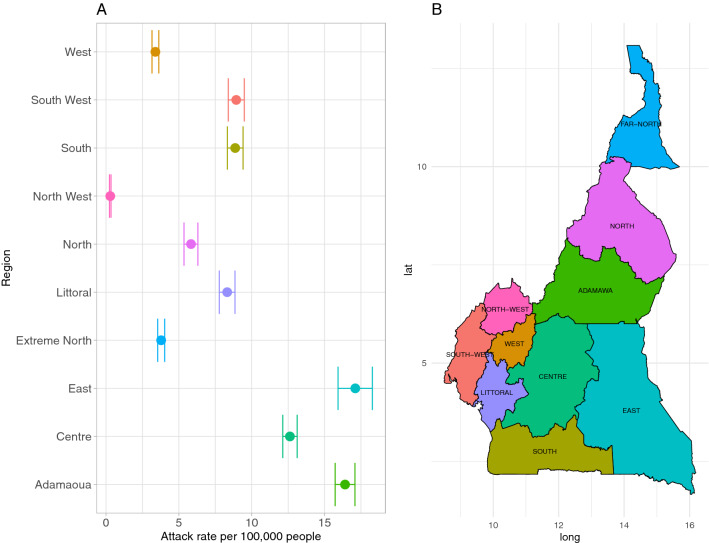
Distribution of ILI reports across the timespan of the study. **(A)** Attack Rate per 100,000 people (mean and 95% confidence interval) for each of the ten regions. **(B)** Map of Cameroon with regions for reference. The map was created in R version 4.0.2.

The weekly number of reported ILI cases and attack rate per 100,000 people from 2012 to 2018, collected by the Cameroon sentinel surveillance system, were obtained from the Ministry of Health (Fig. 1A). The number of reported ILI cases varied across the ten regions in Cameroon. The neighboring East and Adamaoua regions (Fig. 1B) had the highest number of reported cases; 17.1 (95% CI, 15.9–18.3) and 16.4 (95% CI, 15.7–17.1) reports per 100,000 people, respectively. The major economic regions, Littoral and Centre regions which had the most consistent reporting, had different attack rates: 8.3 (95% CI, 7.8–8.9) and 12.6 (95% CI, 12.1–13.1), respectively. There were no distinct regional groupings in reporting patterns.

### Out-of-sample predictions

**Figure 2:**
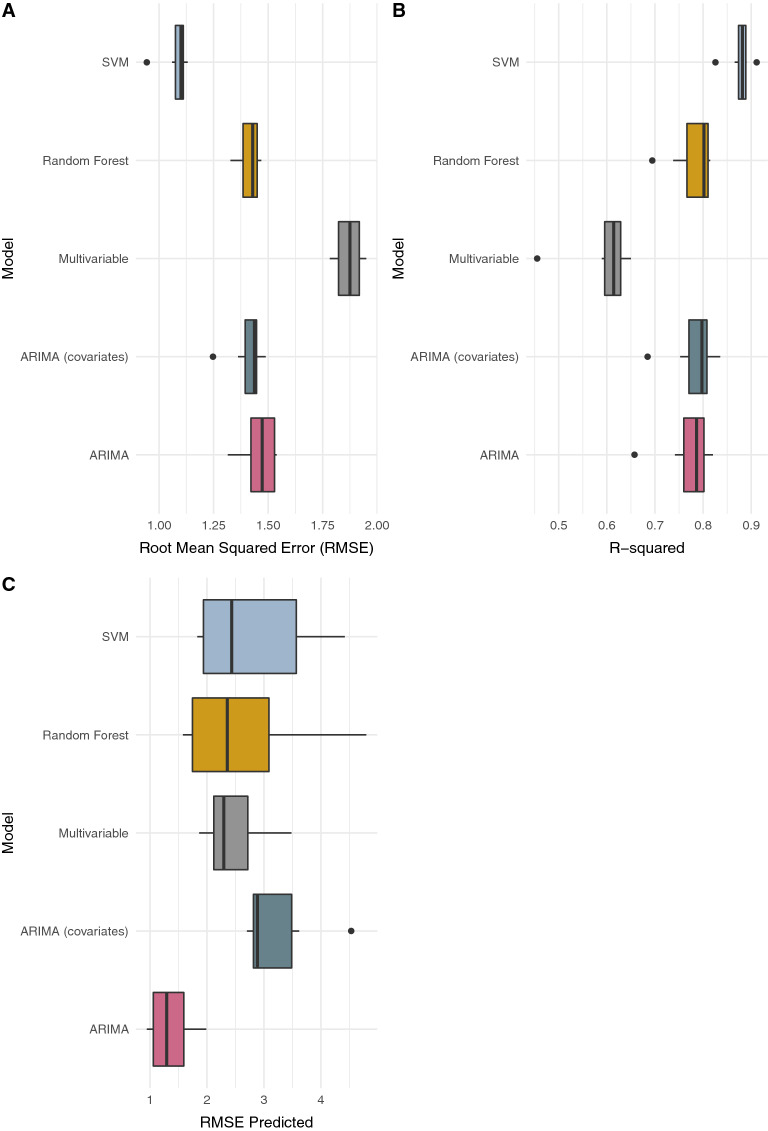
Comparison of models at the national level. **(A)** Root Mean Squared Error (RMSE) of fitted values, **(B)** model R-squared and **(C)** RMSE of out-of-sample predictions. The models are Support Vector Machines Regression (SVM), Random Forest Regression, Multivariable linear regression, and ARIMA with [i.e., ARIMA (covariates)] and without (i.e., ARIMA) search data as explanatory variables.

Out-of-sample predictions are predictions made by a model on data not used for model fitting. It is a cross-validation approach whereby at each modeling step, data excluded from model fitting are used in the model validation (comparing out-of-sample model predictions to data). We estimated the rate of ILI per 100,000 individuals using the following models: random forest regression (RF), support vector regression (SVM), multivariable linear regression and autoregressive integrated moving average (ARIMA) models. The Google search data were used as explanatory variables in all models except one set of ARIMA models, which were used as ‘baseline’ models to compare the model performance with and without Google search data. We used a leave-one-year-out approach (described in the “Methods”) to assess the usefulness of our approach for long term forecasting. Out-of-sample predictions based on the leave-1-year-out approach produced varying results across the regions. At the national level, search terms (SI Table 1) that had correlations of 0.3 or greater with ILI were used as explanatory variables in the models. These included, catarrh, pain, douleur (pain), transpire (sweat), gingembre (ginger), miel (honey), tea, malaria, paludisme (malaria), TB, AIDS, and HIV. SVM had the lowest mean RMSE and the highest mean *R*^*2*^—1.078 and 0.877, respectively (Fig. 2A,B). RF had a mean RMSE of 1.41 and *R*^*2*^ of 0.781. The ARIMA model that did not include the search terms as explanatory variables had comparable RMSE and *R*^*2*^ to Random Forest and the ARIMA model with explanatory variables. The *R*^*2*^ for SVM was statistically significantly different when compared to the multivariable and ARIMA models using the Nemenyi multiple comparison test at the 0.05 significance level. However, the RMSE for SVM was not statistically different when compared to the ARIMA model with covariates and the out-of-sample RMSE was only significantly different between SVM and the ARIMA model without covariates, and between the ARIMA models. Furthermore, the corresponding RMSE range for out-of-sample predictions using SVM was wider when compared to the other models (Fig. 2C); suggesting that SVM might not perform well in long range forecasts.

**Figure 3:**
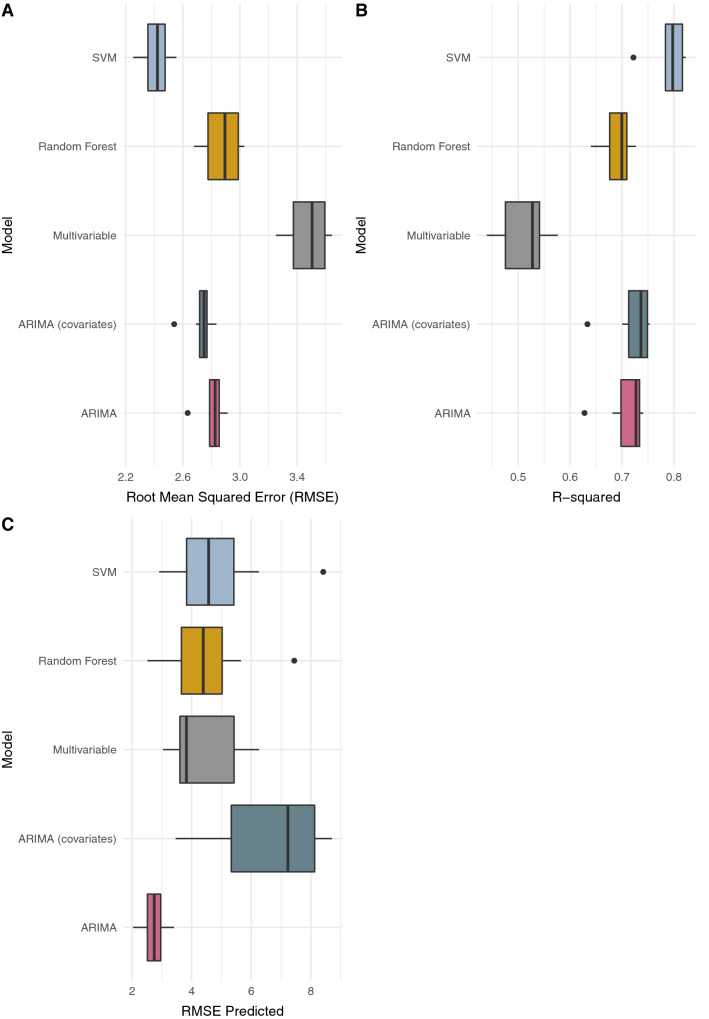
Comparison of models fitted to data for the Littoral region. **(A)** Root Mean Squared Error (RMSE) of fitted values, **(B)** model R-squared and **(C)** RMSE of out-of-sample predictions. The models include Support Vector Machines Regression (SVM), Random Forest Regression, Multivariable linear regression, and ARIMA with [i.e., ARIMA (covariates)] and without (i.e., ARIMA) search data as explanatory variables.

The regional models for Littoral performed similarly to the national models. The mean RMSE for SVM and RF were 2.412 and 2.876, respectively (Fig. 3A). While the corresponding average *R*^*2*^ were 0.791 and 0.691 (Fig. 3B). The mean *R*^*2*^ for the ARIMA models were 0.720 and 0.708 for the models with and without the Google search data, respectively. The respective mean RMSE were also in a similar range—2.73, and 2.80. The mean out-of-sample RMSE (Fig. 3C) was lower for the ARIMA model, and the ranges for models with search terms as explanatory variables were broader. The R^2^ and RMSE for SVM were slightly better than RF (*p* = *0.0115*) and significantly better than multivariable regression (p < 0.005). However, the out-of-sample RMSE was not statistically significantly different. The search terms included in all the models were rhume (cold), cough, douleur (pain), aloe vera, citron (lemon), gingembre (ginger), miel (honey), malaria, TB, AIDS and HIV.

**Figure 4:**
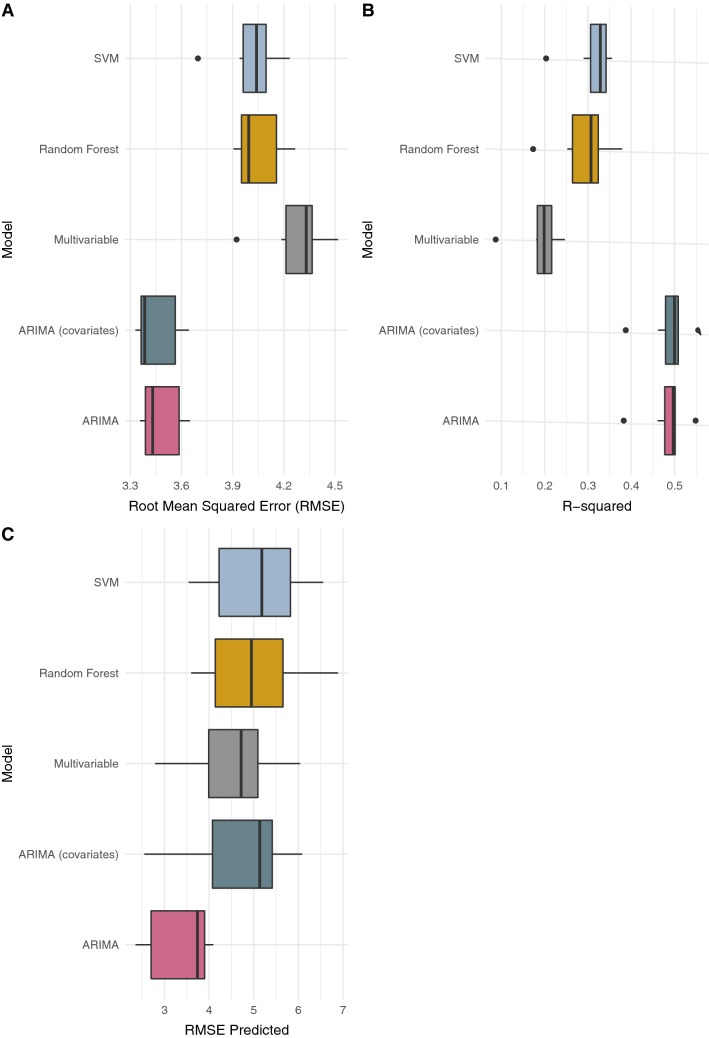
Comparison of models fitted to data for the Centre region. **(A)** Root Mean Squared Error (RMSE) of fitted values, **(B)** model R-squared and **(C)** RMSE of out-of-sample predictions. The models are Support Vector Machines Regression (SVM), Random Forest Regression, Multivariable linear regression, and ARIMA with [i.e., ARIMA (covariates)] and without (i.e., ARIMA) search data as explanatory variables.

In contrast to the Littoral and national models, only three search terms were included in models for the Centre region; gingembre (ginger), lemon and AIDS. The data was noisier, the RMSE was higher for all models (Fig. 4A) and the highest variance explained was 55.5%; much lower compared to the national and Littoral models (Fig. 4B). The autoregressive models performed similarly, while the multivariable linear regression models had the poorest goodness of fit (mean *R*^*2*^ = 0.190) (Fig. 4C). The R^2^ for the ARIMA models were statistically significantly better than the multivariable model. The RMSE for the ARIMA models were also statistically better than the other model and out-of-sample RMSE was statistically better than that for RF and SVM.

These differences across the three regions show variability in data quality and highlight the need for regional models that capture underlying trends in reporting.

### One-week-ahead forecasts

**Figure 5:**
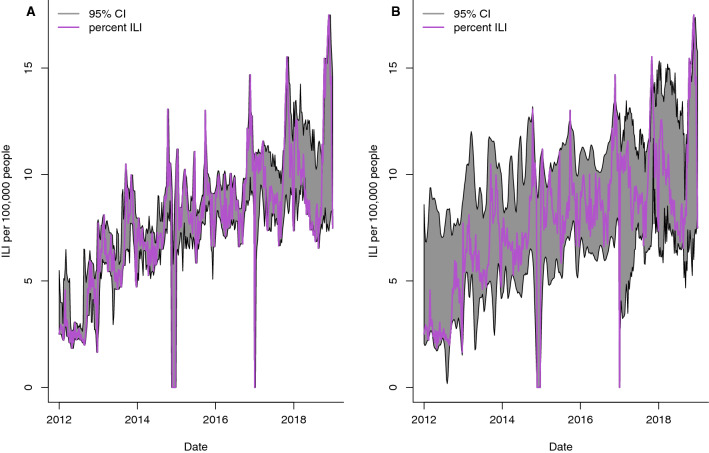
95% Confidence Intervals (CI) for 1-week-ahead predictions of rate of ILI per 100,000 inhabitants at the national level. Models were fit using five years of data; week 1 to 261. Forecasts for the next week, t + 1, were made at week, t. Estimates using **(A)** Random Forest Regression and **(B)** Support Vector Machine Regression (SVM) are presented.

**Figure 6:**
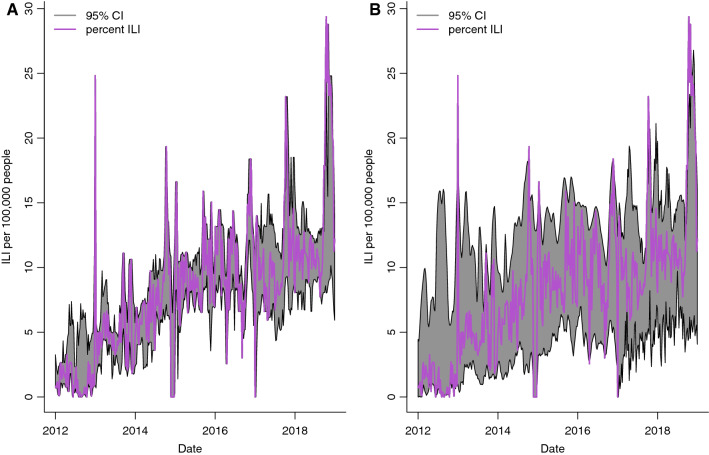
95% Confidence Intervals (CI) for 1-week-ahead predictions of the rate of ILI per 100,000 inhabitants the Littoral region. Models were fit using 5 years of data; week 1 to 261. Forecasts for the next week, t + 1, were made at week, t. Estimates using **(A)** Random Forest Regression and **(B)** Support Vector Machine Regression (SVM) are presented.

The same models used in out-of-sample predictions were also evaluated for one-week ahead forecasts of the rate of ILI per 100,000 individuals. The Google search data were also used as explanatory variables in all models except the baseline model. The correlation between the national rate of ILI per 100,000 individuals and forecasted values were 72.9% and 66.8% using the SVM and Random Forest models, respectively (Fig. 5). The search terms by order of importance in the Random Forest model were as follows: AIDS, catarrh, HIV, paludisme, gingembre, tea, miel (honey), malaria, transpire (sweat), douleur (pain), TB, and pain. Similar results were obtained for the Littoral region, where the correlation between the predicted ILI values and the reported rate of ILI per 100,000 individuals was 80.9% and 60.29% for SVM and Random Forest, respectively (Fig. 6). While the point estimates using the SVM model were more aligned with the reported ILI values, the confidence intervals for Random Forest were more robust. Furthermore, the ARIMA models also had similar performance as the SVM and Random Forest models for both the national (62.4% and 58.9%) and Littoral (71.1% and 71.1%) regions, however, the confidence intervals were not robust. In contrast, the best forecast for the Centre region was based on the ARIMA model using solely ILI data. The correlation between the forecasted values and the ILI data was 44.6%.

## Discussion

Influenza causes considerable disease burden in tropical and subtropical regions. However, despite its global significance, influenza surveillance systems in sub-Saharan Africa are lagging behind those in other regions of the world. National surveillance systems can fill in this gap in monitoring influenza-like illnesses, including emerging viruses, if the systems are well-designed, maintained and regularly evaluated. However, as noted across several countries including the United States, ILI reports from official sources such as, the Centers for Disease Control and Prevention (CDC) can be delayed^9,34^. Digital data sources can be used to supplement data from official sources.

In this study, we have shown that health information seeking trends on the Internet could be accurate predictors of disease trend in sub-Saharan African countries. Our results highlight two important public health opportunities for sub-Saharan Africa. First, health information seeking trends on the Internet are useful for monitoring health information needs in sub-Saharan African countries^27^. So, as mobile phone and Internet usage increases across the continent, data produced using these technologies can provide a supplementary approach for monitoring disease trends. However, the local context is important in both research and interpretation of findings. Search terms commonly associated with specific infectious diseases (such as, influenza or COVID-19) in western countries might not be associated with those diseases in sub-Saharan African countries^35^. For example: ginger, lemon, malaria, and AIDS were shown to be associated with ILI in Cameroon, where this is likely not ne the case in western countries. Second, our study also highlights a need for public health education about influenza and influenza-like illness in Cameroon. Africans, like people in other regions of the world, use the Internet for information seeking. Understanding how different Internet platforms are used can be useful for designing appropriate public health interventions, including disease prevention measures and education on the causes and treatment of diseases^26,27,36^.

Our findings suggest that search data related to influenza symptoms, treatments, natural or traditional remedies as well as infectious diseases with a high burden (i.e., AIDS, malaria, tuberculosis) in sub-Saharan countries could be useful for tracking ILI trends. Specifically, terms such as, cold, catarrh, and pain, and home remedies such as, tea, ginger and honey, were important predictors of ILI trends. Searches for AIDS and HIV might be due to HIV confirmed individuals searching if ILI symptoms are considered HIV symptoms. Studies have shown that individuals prefer using search engines to seek information on stigmatized topics^37^.

Also, our methods perform best in short-term forecasting, but also suggest some usability for long-term forecasting as demonstrated in the leave-1-year-out evaluation. Furthermore, ARIMA models using previous values of ILI to predict current values achieved reasonable accuracy suggesting that previously observed ILI trends can be used for predicting current and future trends. The machine learning regression methods, Random Forest and Support Vector Machines, performed similarly with some minor differences. The confidence intervals in the SVM model were more heavily influenced by variability in the search data and the ILI reports, when compared to the Random Forest estimates. Future studies will focus on developing ensemble approaches involving the consensus of multiple algorithms for real-time forecasting.

Syndromic and pre-diagnostic healthcare data (e.g., school absenteeism, pharmacy sales) in conjunction with statistical methods to monitor and characterize epidemics earlier than traditional public health systems and to provide situational awareness for ongoing outbreaks has been shown to be useful^38,39^. However, clear definitions of disease symptoms are important, especially when many diseases can cause influenza-like symptoms. To accomplish this, interventions including education on the causes and treatments of influenza-like disease are needed. These non-traditional methods can supplement traditional surveillance, however, the need for developing reliable surveillance systems remains. Additionally, consistent data collection procedures and case definition are necessary for evaluation of ILI surveillance systems. ILI cases are most likely severely underreported in Cameroon, and there is not sufficient information to adjust the data accordingly. To use these data and methods in reliable real-time weekly forecasting of ILI cases, these limitations in the data and collection systems need to be addressed.

Another challenge with forecasting ILI trends in subtropical and tropical regions is that seasonality is less defined; some regions experience semi-annual epidemics or annual influenza activity without a well-defined season while others have yearly epidemics that correspond with the rainy season^17,40^. Indoor crowding during the rainy season has been suggested as a possible driver of influenza epidemics in the tropics^21,41^. Moreover, a recent modeling study on Influenza seasonality in the tropics and subtropics showed that absolute humidity and temperature, respectively, have a bimodal and nonlinear effect on influenza transmission in Hong Kong,^42^ whereas previous modeling studies have shown that a unimodal effect of absolute humidity on transmission can accurately forecast the dynamics of seasonal influenza in temperate regions^43,44^. In the temperate regions of the world, influenza has marked seasonal patterns with increased rates during the winter months, followed by low activity during the warmer months^45,46^.

There are also limitations in the use of search data for disease surveillance. For instance, methods addressing spurious spikes in searches due to other factors such as, media coverage or reports of deaths, are needed^47^. This is especially relevant when developing approaches for monitoring emerging diseases such as the COVID-19, where media coverage and digital platforms have influenced the dissemination of information and population response.^48^ Search data is also not always representative of the entire population due to factors such as, variability in internet penetration, access and socioeconomic factors^36,49^. Furthermore, integrating these data with data from other digital sources can help to mitigate limitations inherent in each individual data source^10^.

Despite these limitations, these data have the potential to improve the timely detection of outbreaks and surges in unusual disease activity requiring further public health investigation. A lack of comprehensive health data in most sub-Saharan African countries is a major roadblock to disease control. The most effective approach to mitigating a potential pandemic is to stem disease emergence at its location of origin, the success of which depends on efficient disease surveillance systems. Addressing gaps in surveillance in sub-Saharan Africa should be a priority in our global preparedness and response strategies for influenza and other acute respiratory infections. Data from digital sources could support the development of up-to-date disease surveillance systems.

## Methods

### ILI data

The Cameroon sentinel surveillance system aims to detect the current type of influenza viruses and involves 22 health facilities across the country. The system also involves influenza-like illness surveillance based on symptom definition and is designed for early warning detections of surges. ILI was defined as sudden onset of fever (temperature > 38 °C) and cough or sore throat, with the onset of symptoms within the five days before presentation at a health facility based on the WHO’s Integrated Disease Surveillance and Response (IDSR)^50^. The Cameroon sentinel surveillance system provided a weekly number of cases reported each week to the Cameroon Ministry of Health (MOH) in Yaoundé from January 2012 to December 2018.

### Google Search Data

We obtained the weekly volume of Google searches for 65 terms (see SI Table 1) in both French and English from the Google API at the national level, and for the Centre and Littoral regions from January 2012 to December 2018. Data was sparser for the other regions. The Google search terms consisted of ILI symptoms, home remedies typically used in Cameroon for colds, and inquiries for malaria, HIV/AIDs, and TB. We were particularly interested in home remedies because it captures a context-specific approach to managing ILI symptoms. Next, we excluded all search terms that had zero variance or had a Pearson correlation of less than 0.3, with weekly ILI reports from our data so we could focus our analysis on the most relevant terms. This resulted in three, eleven and twelve terms separately for the Centre region, Littoral region and the whole country. The Google search data represents aggregated anonymized queries that are normalized based on the geographical location and time. The searches are divided by the total volume of searches for that region and specific time range^51^. This suggests that zeroes in the data might not necessarily represent zero searches in a particular time period.

### Statistical analysis

We used statistical and machine learning to models to nowcast ILI trends in Cameroon using weekly reported ILI cases and google search data from January 2012 to December 2018. To assess the potential usefulness of our approach for public health decision making in Cameroon, we performed two sets of analysis. First, we used a cross-validation approach whereby at each modeling step, one year of data is excluded from the fitting and used in the model validation. Since there were seven years of data, this implies seven different models were fitted. Next, the best modeling approach based on the model R^2^ and the root mean squared error (RMSE) were used in one-week ahead forecasting. This allows for forecasting the current reported rate of ILI per 100,000 inhabitants. Because Google search data were sparse for most regions of the country, the analyses were performed only for the Centre and Littoral regions, and the whole country.

We evaluated several multivariable regression models with the search data as explanatory variables and the ILI as the dependent variable. Regression models are traditional statistical models that are widely used to analyze quantitative data and make predictions about future values of the data. Here, we considered a range of robust regression modeling approaches that have been previously used for forecasting influenza trends^11,12,33^: random forest regression, support vector regression, multivariable linear regression and ARIMA models.

Random Forest is an ensemble of regression trees created by using bootstrap samples of the data and random feature selection in tree induction. To construct regression trees each node is split using the best split among a subset of predictors randomly chosen at that node. It is an extension of bagging—bootstrap aggregating—a method for combining several predictors to decrease the variance of the prediction function^52–54^. There are several advantages to random forests including, high accuracy, robustness to overfitting, and estimation of important variables^54^. These are useful for identifying which covariates are most significant in the model^54^. To address bias in Random Forest, we applied the linear model correction conceptually described in Zhang and Lu (2012)^55^.

Regression with Support Vector Machines (SVM) involves mapping the independent variables into a high-dimensional feature space using a linear or non-linear kernel function^56^. SVM regression with the linear kernel is akin to multivariate linear regression models. We used a radial kernel in SVM, since it fitted best to the data trend, and was selected using the tuning function in R^57,58^. The confidence intervals for SVM and RF were obtained using quantile SVM regression and quantile regression forests, respectively^59^.

ARIMA models are represented as ARIMA(p,d,q), where p denotes the autoregressive (AR) order, d the differencing order and q the moving average (MA) order. ARIMA models are widely used in time series forecasting. The model assumes that the time series is stationary^60–62^. The ARIMA models were implemented using the auto.arima function in the forecast package in R^63^ to select the model with the best fit based on the AIC and BIC at each time point . We used the Friedman test and Nemenyi multiple comparison test to compare the R^2^, and RMSE across the models at the 0.05 significance level^64,65^.

The ARIMA, random forest, SVM, and multivariable linear regression models involved fitting ILI to the Google search terms as explanatory variables. To capture the potential delay between the time when an individual with symptoms searches for information online, and when they visit a healthcare facility, we lagged some search terms for up to four weeks in the regression models based on prior examination of the cross correlations between the ILI data and search terms. We also applied LOESS (Local Polynomial Regression Fitting) smoothing to the search terms to capture the overall data trend, while reducing the noise introduced by weeks with zero searches. Similar approaches have been used in studies using digital data for disease modeling^66,67^. The LOESS parameter was set at 0.045.

## Supplementary Information

Supplementary Information.
