# Supplementary material for: Forecasting influenza-like illness trends in Cameroon using Google Search Data
Source: Sci Rep. 2021 Mar 24;11:6713. doi: 10.1038/s41598-021-85987-9 (PMC7991669; doi:10.1038/s41598-021-85987-9)
Supplement: Supplementary file 1 — Supplementary Information. [file 41598_2021_85987_MOESM1_ESM.docx]

**SUPPLEMENTARY INFORMATION**

**Nowcasting Influenza-like Illness Trends in Cameroon using Google Search Data**

Elaine O. Nsoesie^1*^, Olubusola Oladeji^1^, Aristide S. Abah Abah^2^, Martial L. Ndeffo-Mbah^3^

1. Department of Global Health, Boston University School of Public Health, Boston, MA, USA

2. Department of Epidemiological Surveillance, Ministry of Health, Yaoundé, Cameroon

3. Department of Veterinary Integrative Biosciences, College of Veterinary Medicine and Biomedical Sciences, Texas A & M University, Texas, USA

* Corresponding author: Elaine Nsoesie, Department of Global Health, Boston University School of Public Health, 801 Massachusetts Ave, Crosstown Center 3^rd^ Floor, Boston, MA 02119; onelaine@bu.edu

**Table 1: Google Search Terms**

| Term Group | English | French |
| --- | --- | --- |
| Symptoms | catarrh, chills, cough, fever, headache, pain, muscle ache, muscle pain, sore throat, throat pain, sweat, fatigue, stuffy nose, body ache, tiredness, rhinorrhea, myalgia, red eyes | catarrhe, frisson, toux, fievre, mal de tete, douleur, douleur musclaire, gorge irritee, transpire, yeux rouges, malaise, courbatures, myalgie, fatigue |
| Cold home remedies | aloe vera, ginger, honey, lemon, onion, shea butter, steam, tea, ginger tea, pepper soup, hot bath, mentholatum | citron, citronnelle, gingembre, miel, oignon |
| Disease | flu, influenza, cold, HIV, malaria, TB, AIDS, tuberculosis | grippe, rhume, tuberculose, paludisme, SIDA |
| Other | health insurance |  |

**METHODS**

**Multivariate Linear Regression (MLR):** Given a dataset with *n* independent variables and *m* observations, the MLR model can be written as y = ƒ(*x*) = *W*$\cdot$*X* +b where *W* represents the vector of the coefficients, *X* represents the vector of the independent variables, and b is the intercept. To estimate the best fit, we minimize the sum of the squared errors:

$\sum_{i=1}^{m} \left( y_{i}-\left( \hat{W}\cdot X_{i}+\hat{b} \right) \right)^{2}$where *i* represents the *i* th observation.

**Support Vector Machines (SVM):** Regression with SVM differs from that of MLR in the underlying theoretical settings. The basic idea of regression methods is to construct an optimal regression hyperplane with n-1 dimensions that best fits the data in an n-dimensional space. MLR algorithm fits a model using the least mean squares approach to define the linear hyperplane and the regression based on a least mean squares approach is greatly affected by outliers. In the SVR method, these problems are resolved by 1) using integrating kernel functions to add more dimensions to lower dimensional space or add nonlinearity to the model; and 2) introducing user-specified parameters to control the trade-off of prediction errors and flatness of the regression plane.^1^

For example, when the correlation between *X* and *y* is linear, the form of the SVR algorithm is similar to that of MLR: y = ƒ(*x*) = *W*$\cdot$*X* +b. However, the SVR method has two additional parameters: *C* and ε. The parameter *C* is introduced to adjust the error sensitivity of the training data in order to avoid over-fitting, while the second parameter ε is the regularization constant, which controls the flatness of the final model^2^. The goal of SVR is then to determine an optimal function that has less than ε deviation from the target values for the training data, so that we do not count errors that are less than ε, and at the same time the regression hyperplane is as flat as possible. Here the objective is to minimize

$\sum_{i=1}^{m} \left( y_{i}-\left( \hat{W}\cdot X_{i}+\hat{b} \right) \right)^{2}+C\sum_{j=1}^{n} |W_{j}^{2}|$where *j* represents the *j* th variable.

When the correlation between *X* and *y* is nonlinear, SVM allows application of nonlinear regression by using kernel functions such as polynomial, Gaussian, Radial Basis Function, and Sigmoid kernel.

**Random Forest Regression:** Random Forest is an ensemble learning method involving the construction of several de-correlated decision trees at training and the predictions of the individual trees are averaged.^3–5^ It is an extension of bagging – bootstrap aggregating – a method for combining several predictors to decrease the variance of the prediction function.

The essential idea in bagging is to average many noisy but approximately unbiased models, and hence reduce the variance. Trees are ideal candidates for bagging, since they can capture complex interaction structures in the data, and if grown sufficiently deep, have relatively low bias. Moreover, since each tree generated in bagging is identically distributed, the expectation of an average of *B* such trees is the same as the expectation of any one of them. This means the bias of bagged trees is the same as that of the individual trees, and the only hope of improvement is through variance reduction. This is in contrast to boosting, where the trees are grown in an adaptive way to remove bias. The idea in random forests (see algorithm below) is to improve the variance reduction of bagging by reducing the correlation between the trees. This is achieved in the tree-growing process through random selection of the input variables. After *B* such trees ${\{T_{b}\}}_{B}^{1}$ are grown, the random forest regression predictor is $f(x)=\frac{1}{B}\sum_{b=1}^{B} T_{b}(x)$

*Random Forest Regression algorithm*:

1. For *b* = 1 to *B*:
2. Draw a bootstrap sample Z^∗^ of size *N* from the training data.
3. Grow a random-forest tree $T_{b}$to the bootstrapped data, by recursively repeating the following steps for each terminal node of the tree, until the minimum node size $n_{min}$is reached.

i. Select $m$ variables at random from the $p$variables.

ii. Pick the best variable/split-point among the $m$.

iii. Split the node into two daughter nodes.

1. Output the ensemble of trees ${\{T_{b}\}}_{B}^{1}$.

To make a prediction at a new point $x$:

Regression: $f(x)=\frac{1}{B}\sum_{b=1}^{B} T_{b}(x)$

**REFERENCE**

1. Drucker, H., Burges, C. J., Kaufman, L., Smola, A. J. & Vapnik, V. Support vector regression machines. in *Advances in neural information processing systems* 155–161 (1997).

2. Chapelle, O., Vapnik, V., Bousquet, O. & Mukherjee, S. Choosing Multiple Parameters for Support Vector Machines. *Mach. Learn.* **46**, 131–159 (2002).

3. Breiman, L. Bagging predictors. *Mach. Learn.* **24**, 123–140 (1996).

4. Breiman, L. Random Forests. *Mach Learn* **45**, 5–32 (2001).

5. Hastie, T., Tibshirani, R. & Friedman, J. *The elements of statistical learning*. (2009).
